# Role of alarmin cytokines and microRNAs in the host-schistosome interaction

**DOI:** 10.12688/f1000research.15695.1

**Published:** 2018-09-28

**Authors:** Xing He, Weiqing Pan

**Affiliations:** 1 Department of Tropical diseases, Second Military Medical University, Shanghai, 200433, China

**Keywords:** alarmin cytokine, microRNA, schistosomiasis

## Abstract

Schistosomiasis is a serious but neglected tropical infectious disease, afflicting more than 240 million people in 78 countries. Lack of an effective vaccine and obscuring disease mechanism could be the main hurdles to effectively control and eradicate this disease. A better understanding of the host–schistosome interaction is the key to clearing these hurdles. Recently, accumulating evidence shows that alarmin cytokines and microRNAs (miRNAs) are crucial regulators in the host–schistosome interaction. Alarmin cytokines are proven to be potent mechanisms driving type 2 immunity, which is the central disease mechanism of schistosomiasis. MiRNA deregulation is a hallmark of a variety of human diseases, including schistosomiasis. In this review, we summarize the research advances on the role of alarmin cytokines and miRNAs in the host–schistosome interaction.

Schistosomiasis is one of the most prevalent, but neglected, tropical infectious diseases, afflicting more than 240 million people in 78 countries, including children and young adults ^1^. It is caused by trematode parasites of the genus *schistosoma*, of which three are known to cause severe disease in humans, namely *Schistosoma mansoni* ( *S. mansoni*), *Schistosoma japonicum*, and *Schistosoma haematobium*. *S. mansoni* and *S. japonicum* result in intestinal schistosomiasis, whereas *S. haematobium* is responsible for urinary schistosomiasis. Current strategies to control this disease rely heavily on the administration of praziquantel ^2^. However, its widespread use may result in the development of drug resistance ^3^. Vaccination is a good strategy to control or eradicate this disease; however, an effective vaccine is still lacking because of our limited knowledge of the immunological mechanisms associated with the elimination of the pathogen ^4^. In addition, hepatic fibrosis is the primary cause of morbidity and mortality from schistosomiasis, but the disease mechanism remains elusive, and effective intervention is lacking ^1^. A better understanding of the mechanism of the host–schistosome interaction is the key to solving these problems. Accumulating evidence shows that alarmin cytokines and microRNAs (miRNAs) are crucial regulators in the host–schistosome interaction. In this review, we summarize the research advances in these fields.

## Role of alarmin cytokines in the host–schistosome interaction

Schistosomiasis is an immune pathological disease. The type 2 immune response, characterized by the T helper 2 (Th2) cell-associated cytokines, such as interleukin 4 (IL-4) and IL-13, is the central regulator of disease progression in schistosomiasis ^5^. However, the signals that drive the type 2 immune response after infection remain largely unknown ^6^. Numerous studies have highlighted that tissue damage, which induces the release of alarmin cytokines, including IL-25, IL-33, and thymic stromal lymphopoietin (TSLP), is a potent mechanism driving type 2 immunity ^7^, particularly in the context of helminth infection ^8^. TSLP regulates dendritic cells, basophils, mast cells, monocytes, natural killer T cells, and type 2 innate lymphoid cells (ILC2s) ^9^, whereas IL-25 and IL-33 exhibit similar Th2-promoting activity largely by stimulating ILC2s, basophils, mast cells, and eosinophils ^10, 11^. The role of alarmin cytokines in schistosomiasis has been intensively studied; however, published results have been inconsistent ^12– 15^.

Serum IL-33 was observed to be markedly increased in patients infected with *S. japonicum* ^16^, whereas serum IL-33 and intracellular IL-13 in eosinophils were elevated in patients infected with *S. haematobium* after chemotherapy ^17^. In addition, serum soluble ST2, a decoy receptor of IL-33, was significantly elevated in patients with end-stage *S. japonica* infection, whereas membrane ST2 expression was obviously increased in the fibrotic liver tissues ^18^. In addition, ST2 genetic variants were strongly associated with serum soluble ST2 levels ^18^. In a mouse model of schistosomiasis, ST2 deficiency or blockade of IL-33 using soluble ST2 treatment or neutralizing antibodies showed significantly less Th2-mediated pathology, including marked decreases in granuloma and fibrosis formation, or Th2 cytokine production ^12, 13, 19^. Decreased pulmonary collagen deposition and granuloma size were also observed in mice deficient in IL-25 or its receptor following *S. mansoni* egg challenge ^14^. In addition, IL-33 regulated hepatic type 2 pathology after schistosome infection by promoting the production of type 2 macrophages (M2) ^20^. Our group found that hepatic stellate cells (HSCs) were the primary source of IL-33 and that ILC2s were the primary source of IL-13 in the infected mouse livers ^21^. However, two other groups proved that IL-33– or IL-25–deficient mice exposed to schistosome eggs or cercariae showed no significant differences in granuloma size and collagen deposition ^15, 22^. Instead, marked reductions in granuloma size and fibrosis extent were observed when IL-25, IL-33, and TSLP were simultaneously disrupted ^15^. In addition, primary, but not secondary, granulomatous inflammation in the lungs challenged with eggs was reduced in TSLP receptor–deficient mice, and hepatic fibrosis instead of granuloma was reduced in these mice ^23^.

These studies seemed to imply that the roles of TSLP, IL-25, and IL-33 in the initiation of type 2 immunity induced by schistosome infection were redundant, and single alarmin cytokine had little impact on the course of schistosome infection. Given the potential therapeutic value of these alarmin cytokines in controlling Th2 pathology, further research is needed to determine their roles in schistosomiasis. It appears that the inconsistency in the published results might be caused by the differences in intervention time, especially for IL-33. When IL-33 was depleted in the embryo stage, the role of IL-33 might be compensated for by other factors; however, when IL-33 was neutralized during disease progression, the role of IL-33 in disease progression became more obvious.

## Role of microRNAs in the host–schistosome interaction

Over the last decade, miRNAs have emerged as important regulators of human diseases ^24^. MiRNAs are endogenous, small non-coding RNAs that negatively regulate post-transcriptional gene expression through binding with partial complementarity to their target mRNA sequences ^25^. In schistosomiasis, miRNAs have been increasingly studied for their potential roles in host–parasite interactions.

### Host microRNA deregulation during infection

**Figure 1.**
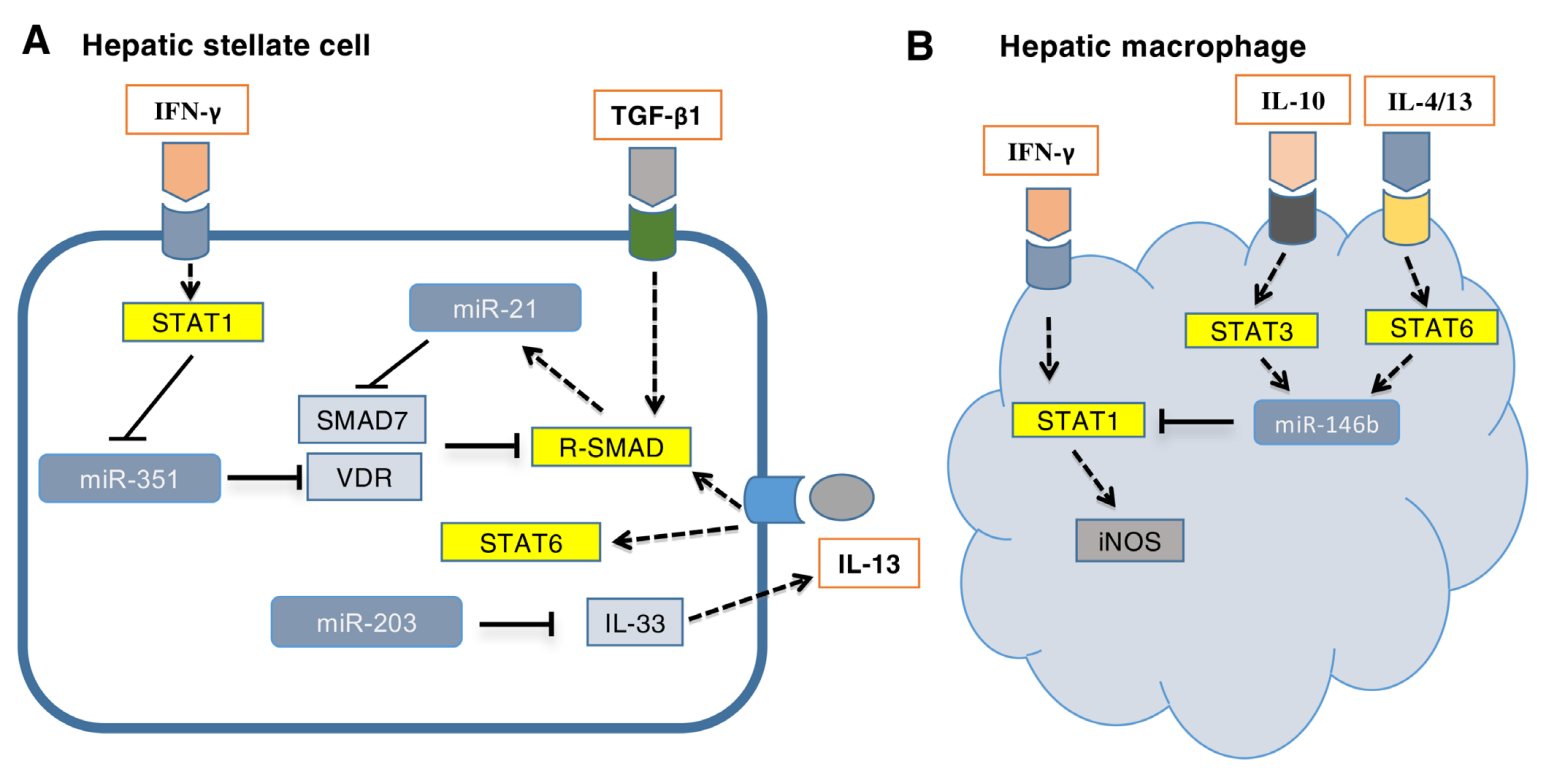
Role and regulation of host microRNAs in hepatic schistosomiasis. ( **A**) Transforming growth factor-beta 1 (TGF-β1) and interleukin-13 (IL-13) are the primary cytokines responsible for fibrosis induced by schistosome infection by activation of SMAD pathway. In hepatic stellate cells, miR-21, induced by TGF-β1 and IL-13, prompted TGF-β1/SMAD and IL-13/SMAD signaling to induce fibrogenic effects by relieving the inhibitory effect of SMAD7 in the SMAD pathway. MiR-351 was negatively regulated by interferon-gamma (IFN-γ) via activation of the STAT1 pathway and promoted fibrogenesis by targeting vitamin D receptor (VDR), a newly identified negative regulator of the SMAD pathway. Moreover, infection-induced downregulation of miR-203 expression resulted in hepatic fibrosis by relieving the inhibition of IL-33, which elevated the expression of IL-13. ( **B**) In hepatic macrophages, various T helper 2 (Th2) cytokines, such as IL-4, IL-13, and IL-10, promoted the transcription of miR-146b by activating STAT3/6, and elevated miR-146b inhibited the IFN-γ–induced differentiation of macrophages into M1 cells by targeting STAT1. iNOS, inducible nitric oxide synthase.

MiRNA deregulation is a hallmark of a variety of human diseases, including infectious diseases. Identification of host-deregulated miRNA during infection may uncover novel disease mechanisms or potential therapeutic targets. Alterations in host miRNAs following schistosome infection have been studied extensively. Hepatic fibrosis is the primary cause of morbidity and mortality from human schistosomiasis, and HSCs are the main effector cells for hepatic fibrosis ^26^. Via a miRNA microarray, a series of host miRNAs have been identified that were deregulated during the progression of hepatic fibrosis in a mouse model of *S. japonicum* infection. Importantly, adeno-associated virus 8 (AAV8)-mediated inhibition of those host miRNAs, such as miR-21 and miR-351, or elevation of miR-203 significantly protected hosts from lethal schistosomiasis via attenuation of hepatic fibrosis ^21, 27, 28^. HSCs were target cells of these three crucial host miRNAs. In HSCs, miR-21 was induced by transforming growth factor-beta 1 (TGF-β1) and IL-13, the primary cytokines responsible for fibrosis induced by schistosome infection ^29^. In addition, elevated miR-21 prompted TGF-β1/SMAD and IL-13/SMAD signaling to induce fibrogenic effects by relieving the inhibitory effect of SMAD7 in the SMAD pathway. MiR-351 levels are elevated during the infection and the increased miR-351 promoted the activation of HSCs by targeting the vitamin D receptor, a newly identified negative regulator of the SMAD pathway ^30^. Moreover, the schistosome infection downregulated miR-203 expression, which relieved the inhibition of IL-33, and sequentially elevated levels of IL-33 were released into the liver tissue, which stimulated the proliferation and IL-13 production of hepatic ILC2s ( Figure 1A). In addition, Zhu *et al.* found that miR-454 promoted the activation of HSCs after infection by targeting SMAD4 ^31^. Importantly, there is evidence that host miRNAs played an important role in regulating hepatic fibrosis in humans infected with *S. japonicum* ^32^.

Schistosomiasis is an immune pathological disease, and various types of immune cells, especially macrophages and T cells, play an important role in disease progression. We found that elevated miR-146b inhibited the interferon-gamma (IFN-γ)-induced differentiation of macrophages into M1 cells by targeting STAT1 ( Figure 1B) ^33^. Kelada *et al.* showed that miR-182 critically prevented IL-2 production in Th2-associated regulatory T (Treg) cells ^34^. These studies highlighted that miRNAs are crucial regulators in the initiation and progression of schistosomiasis, and targeting the deregulated miRNA is a potential therapeutic intervention for this chronic disease.

### Regulation of host microRNA expression by schistosome infection

The interplay among various cytokines, including both Th1 and Th2 cytokines, has a crucial role in the initiation and progression of schistosomiasis. IL-13 and TGF-β1 are the effector cytokines of hepatic fibrosis induced by schistosome, whereas IFN-γ has anti-fibrotic activity in this disease ^29^. IL-4 is responsible for the formation of egg granuloma, and IL-10 is the main negative regulator of pathology ^6^. Interestingly, these cytokines also play a crucial role in the regulation of host miRNA expression during schistosomiasis. IL-13 and TGF-β1 additively upregulated the expression of miR-21 in HSCs by activating SMAD proteins, which promotes the maturation of miR-21 ^27^. IFN-γ inhibited the expression of miR-351 in HSCs through a pathway dependent on the transcription factor STAT1 to induce the expression of interferon regulatory factor 2 (IRF2), which binds to the promoter of *pre-miR-351* to inhibit its transcription ^28^ ( Figure 1A). Various Th2 cytokines, including IL-4, IL-10, and IL-13, could induce macrophages to express miR-146b by activating STAT3/6, which bind to the promoter of the *pre-miR-146b* gene and initiate its transcription ^33^ ( Figure 1B). In Treg cells, IL-4 regulated miR-182 by inducing cMaf, an IL-4–regulated transcription factor ^34^. These studies uncovered the mechanisms by which cytokines regulate disease progression and further highlighted their crucial roles in this disease.

### Schistosome microRNAs and their cross-species regulation of host genes

The availability of parasite genome sequences, combined with advances in RNA sequencing, has paved the way to identify novel miRNAs in schistosomes. The presence of miRNAs in *S. japonicum* was first reported by our group through cloning and sequencing a small (18- to 26-nucleotide) RNA cDNA library from adult worms ^35^. Subsequently, detailed information of miRNA expression in this parasite was generated through the analysis of small RNA libraries from particular developmental stages ^36^. As of 25 June 2018, a search for *S. japonicum* and *S. mansoni* indicated that 171 pre-miRNAs are currently annotated in miRBase ( www.mirbase.org/search.shtml). The expression of many of these parasite miRNAs is stage-, gender-, or cell type-specific, implying their crucial roles in parasite development and sex maturation ^37^. Targeting these miRNAs and their regulated genes or pathways should be a novel strategy for disease control, but it is difficult to implement this strategy because of limited genetic manipulation for the parasite ^38^. However, miRNAs are a highly conserved group of small RNA molecules, expressed by most organisms, and have similar mechanisms of miRNA function. Recent studies showed that miRNAs could be released from pathogens, such as bacteria and parasites, living in the hosts ^39– 41^. These cell-free miRNAs are very stable in the host body fluids and can be absorbed by distant host recipient cells, which suggest that pathogen-derived miRNAs can regulate the function of host cells in a cross-species manner. This new manner of host–parasite interaction has been validated in an animal model infected with *Heligmosomoides polygyrus* ^42^. The miRNAs contained in parasite exosomes suppressed type 2 innate immunity in mice. Accumulating evidence shows that all stages of the schistosome could secrete exosome-like vesicles that contain numerous parasite miRNAs and that these vesicles could transport their cargo miRNAs to host cells, where the parasite-derived miRNAs regulated host gene expression, thereby exerting their effects on the occurrence and progression of host disease during infection ^43– 45^.

## Conclusions

The type 2 immune response is the central regulator of disease progression in schistosomiasis. The role of alarmin cytokines in schistosomiasis has been intensively studied; however, the results from different studies have been inconsistent. Further research is needed to address these inconsistences. Host miRNAs are crucial regulators of disease progression and targeting host-dysregulated miRNAs is a potential strategy to treat this chronic disease. Parasite infection induces the expression of certain cytokines that regulate the expression of host miRNAs through various signaling pathways, which contribute to modulating the occurrence and progression of host diseases. Parasite-derived miRNA-mediated cross-species regulation of host genes might promote various disease processes or strengthen host resistance to the diseases. These studies have already broadened our understanding of the mechanisms of host–schistosome interaction and may be translated into new therapeutic targets.
